# Rapid Purification and Procoagulant and Platelet Aggregating Activities of Rhombeobin: A Thrombin-Like/Gyroxin-Like Enzyme from *Lachesis muta rhombeata* Snake Venom

**DOI:** 10.1155/2013/903292

**Published:** 2013-08-24

**Authors:** Frank Denis Torres-Huaco, Cláudio C. Werneck, Cristina Pontes Vicente, Talita Vassequi-Silva, Ana Cláudia Coelho Nery-Diez, Camila B. Mendes, Edson Antunes, Sérgio Marangoni, Daniela C. S. Damico

**Affiliations:** ^1^Department of Biochemistry, Institute of Biology, University of Campinas (UNICAMP), P.O. Box 6109, 13083-970 Campinas, SP, Brazil; ^2^Department of Structural and Functional Biology, Institute of Biology, State University of Campinas (UNICAMP), P.O. Box 6109, 13083-865 Campinas, SP, Brazil; ^3^Department of Pharmacology, State University of Campinas (UNICAMP), P.O. Box 6109, 13083-887 Campinas, SP, Brazil

## Abstract

We report a rapid purification method using one-step chromatography of SVSP Rhombeobin (LMR-47) from *Lachesis muta rhombeata* venom and its procoagulant activities and effects on platelet aggregation. The venom was fractionated by a single chromatographic step in RP-HPLC on a C8 Discovery BIO Wide Pore, showing high degree of molecular homogeneity with molecular mass of 47035.49 Da. Rhombeobin showed amidolytic activity upon BA**ρ**NA, with a broad optimum pH (7–10) and was stable in solution up to 60°C. The amidolytic activity was inhibited by serine proteinase inhibitors and reducing agents, but not chelating agents. Rhombeobin showed high coagulant activity on mice plasma and bovine fibrinogen. The deduced amino acid sequence of Rhombeobin showed homology with other SVSPs, especially with LM-TL (*L. m. muta*) and Gyroxin (*C. d. terrificus*). Rhombeobin acts, *in vitro*, as a strong procoagulant enzyme on mice citrated plasma, shortening the APTT and PT tests in adose-dependent manner. The protein showed, “*ex vivo*”, a strong defibrinogenating effect with 1 *µ*g/animal. Lower doses activated the intrinsic and extrinsic coagulation pathways and impaired the platelet aggregation induced by ADP. Thus, this is the first report of a venom component that produces a venom-induced consumptive coagulopathy (VICC).

## 1. Introduction

Snakes of the genus *Lachesis*, commonly known as bushmasters, or surucucu in Brazil, can be found in the tropical forests of Central and South Americas. There are three different *Lachesis* species: *L. stenophrys*, *L. melanocephala* (both found in Central America), and *L. muta*. In Brazil, two subspecies can be found: *L. muta muta* in the Amazon Forest and *L. muta rhombeata* in the Atlantic Forest along the eastern coast of Brazil [1].

Poisoning by *Lachesis* is characterized by local damage, pain, edema, hemorrhage, and myonecrosis, as well as systemic complications, such as coagulation disorders, hemolysis, neurotoxicity, renal failure, diarrhea, hypotension, and bradycardia, among others [2–5]. *Lachesis m. rhombeata* crude venom shows lower lethal and hemorrhagic activities when compared with *L. m. muta* venom, while both venoms induce similar edema-forming and myotoxic activities. However, *L. m. rhombeata* venom exhibits higher coagulant and defibrinogenating effects [6].

Snake venom serine proteinases (SVSPs) belong to the trypsin S1 family of SA clan, the largest family of peptidases [7, 8]. SVSPs are among the best-characterized venom enzymes affecting the haemostatic system. They act on a variety of components of the coagulation cascade, on the fibrinolytic and kallikrein-kinin systems, and on platelets to cause an imbalance of the haemostatic system of the prey [8]. 

So far, two serine proteinases were isolated from *Lachesis m. rhombeata*: a thrombin-like/gyroxin-like enzyme denominated LMR-47 [9] and LMR kininogenin [10]. However, none of these proteins were fully characterized biochemically or had their effects studied over the haemostatic system. Since the *L*. *m*. *rhombeata* crude venom induces strong effects over the haemostatic system, it was decided to investigate the participation of the SVSP LMR-47 in those effects induced by the envenomation of this snake species.

 In this study, we report a one-step method of purification, prediction of primary structure, and “*in vivo*” effects over the coagulation cascade as well as over the platelet aggregation of SVTLE LMR-47, which will suggest its possible role in relation to the effects on the haemostatic system of *Lachesis m. rhombeata* crude venom. Also, we propose to rename this protein as Rhombeobin according to its thrombin-like activity.

## 2. Materials and Methods

### 2.1. Venom and Reagents

*Lachesis m. rhombeata* venom was gently donated by Dr. Rodrigo C. G. de Souza from Serra Grande Center for *Lachesis muta *Breeding, with IBAMA Authorization no. 24945-1. All chemicals and reagents were of analytical or sequencing grade. Bovine fibrinogen was purchased from Amour Pharmaceutical, CA, USA. 

### 2.2. Animals

Four-five-week male Swiss mice were supplied by the Animal Services Unit of the State University of Campinas (UNICAMP). Mice were housed at room temperature on a 12 h light/dark cycle and had free access to food and water. All procedures were performed according to the general guidelines proposed by the Brazilian Council for Animal Experimentation (COBEA) and were approved by the university's Committee for Ethics in Animal Experimentation (CEEA/UNICAMP) no. 1790-1.

### 2.3. Venom Fractionation

Five mg of crude venom of *L. m. rhombeata* was dissolved in 0.2 mL of solvent A (0.1% (v/v) trifluoroacetic acid). The resulting solution was clarified by centrifugation, and the supernatant was further submitted to a reversed-phase chromatography on a C8 Discovery BIO Wide Pore (25 cm × 4.6 mm × 10 *μ*m). Fractions were eluted using a stepwise gradient of solvent B (66% acetonitrile in solvent A) (0%–55% B for 26 min, followed by 55%–65% B over 20 min, and 65%–100% B over 14 min). The flow rate of 1.0 mL/min was constant, and the resulting fractions were manually collected. The elution profile was monitored at 280 nm, and the collected fractions were lyophilized and conserved at −20°C. The homogeneity of the final material was assessed by a rechromatography on the same column with a linear gradient (0%–100%) of solvent B.

### 2.4. SDS-PAGE

Sodium dodecyl sulfate-polyacrylamide gel electrophoresis (SDS-PAGE) was carried out on purified Rhombeobin according to Laemmli [20]. The molecular mass markers used were (in kDa)the following: phosphorylase B, 94; albumin, 67; ovalbumin, 43; carbonic anhydrase, 30; soybean trypsin inhibitor, 20; and lysozyme, 14.

### 2.5. Determination of Protein Concentration

Protein concentration was determined by the method of Bradford [21] and standardized with bovine serum albumin.

### 2.6. Amidolytic Activity and Determination of Kinetic Parameters

Amidolytic activity was measured using the synthetic substrate N-benzoyl-L-arginine *ρ*-nitroanilide (BA*ρ*NA) modified for 96-well plates. The standard assay mixture contained 50 *μ*L of buffer (10 mM Tris-HCl, pH 8.0, 10 mM CaCl_2_, and 100 mM NaCl), 200 *μ*L of substrate solution (1 mM), 15 *μ*L of water, and 5 *μ*L of fractions or enzyme (Rhombeobin) in a final volume of 270 *μ*L. The reaction was carried out in a VERSAMAX microplate reader (Molecular Devices Corporation, Sunnyvale, CA, USA) for 30 min at 37°C, with the absorbance being read at 410 nm. The results were expressed as the initial velocity of the reaction (*V*_0_) calculated based on the amount of *ρ*-nitroaniline released [22]. The temperature and pH effect on amidolytic activity of Rhombeobin was examined by incubating the overall mixture reaction at different temperatures (10–80°C) and pH (5–11). At different BA*ρ*NA concentrations (0.0095–10 mM), the *K*_*m*_ and *V*_max⁡_ of Rhombeobin were determined under optimal pH and temperature. The effects of inhibitors EDTA (5 mM), EGTA (5 mM), PMSF (2 mM), SBT-I (1 mg/mL), and DTT (5 mM) were tested by preincubating the sample with these compounds for 30 min at 37°C prior to the standard test. The inhibition results were expressed as percentage of residual activity.

### 2.7. Fibrinogenolytic Activity

Fibrinogenolytic activity was determined by mixing 10 *μ*L of Rhombeobin with 200 *μ*L of bovine fibrinogen solution (2 mg/mL in 10 mM Tris-HCl, pH 7.4, 10 mM CaCl_2_, and 100 mM NaCl) at 37°C, at different incubation times (5 min–24 h) and different concentrations of enzyme (0.632–10 *μ*g). The reaction was stopped with 150 *μ*L of denaturizing solution (Tris-HCl 0.05 M, pH 6.5, urea 10 M, b-mercaptoethanol 10% (v/v), glycerol 10% (v/v), and bromophenol blue 0.05% (v/v)), incubated for 15 minutes at room temperature. Fibrinogenolytic activity was evaluated by SDS-PAGE using 12.5% polyacrylamide gels as described by Laemmli [20].

### 2.8. Determination of Minimum Coagulant Dose (MCD)

The MCD over bovine fibrinogen (MCD-F) or plasma (MCD-P) of Rhombeobin was determined according to Theakston and Reid [23]. Briefly, 0.25 to 10 *μ*g of Rhombeobin (20 *μ*L PBS) were added to 200 *μ*L of bovine fibrinogen solution (2 mg/mL) or mice citrated plasma at 37°C. The solutions were mixed thoroughly, and the clotting times were recorded. The MCDs for bovine fibrinogen and plasma were calculated by plotting Log of Rhombeobin concentration versus Log of the clotting times. The MCD was defined as the least amount of toxin (in mg of dry weight per liter of test solution) that clots citrated plasma or bovine fibrinogen solution in 60 seconds at 37°C. 

### 2.9. Determination of the Molecular Mass of the Purified Protein by Mass Spectrometry

An aliquot (4.5 *μ*L, 0,1% formic acid) of Rhombeobin (10 *μ*g) was injected by C18 (100 mm × 100 mm) RP-UPLC (nanoACQUITY UPLC, Waters) coupled with nanoelectrospray tandem mass spectrometry on a Q-Tof Ultima API mass spectrometer (MicroMass/Waters) at a flow rate of 600 nL/min. The gradient was 0%–50% acetonitrile in 0.1% formic acid over 45 min. The instrument operation and the processing parameters were set according to Damico et al. [24]. 

### 2.10. Sequencing Procedures

The reduced (5 mM dTT for 25 min at 56°C) and alkylated (14 mM iodoacetamide, 30 min) protein was digested with trypsin (Promega-Sequencing Grade Modified) or SV-8 (Promega-Sequencing Grade Modified). The digestion procedures, the peptide separation, and the mass analysis of the resulting peptides were made following the methodology described by Damico et al. [24].

Raw data files from LC-MS/MS runs were processed using Masslynx 4.1 software package (Waters) and were analyzed using the MASCOT search engine version 2.3 (Matrix Science, Ltd.) against the snakes database, using the following parameters: peptide mass tolerance of ±0.1 Da, fragment mass tolerance of ±0.1 Da, oxidation as variable modification in methionine, and trypsin as enzyme.

### 2.11. Evaluation of Gyroxin Syndrome

Rhombeobin gyroxin-like activity was determined according to Seki et al. [25]. Briefly, 25 *μ*L of enzyme (1–20 *μ*g protein in PBS) was injected iv. into adult mice weighing 25 ± 2 g. After injection, it was determined whether the animals showed the characteristic equilibrium loss and, eventually, typical rolling movements as described by Barrabin et al. [26]. The animals were observed 24 h after injection in order to rule out the contamination of the purified fraction with lethal toxins.

### 2.12. Activated Partial Thromboplastin Time (APTT) and Prothrombin Time (PT)

Male Swiss mice were anesthetized (xylazine, 2%–16% mg/kg; ketamine, 10%–100% mg/kg), and blood was collected from cava vein in citrate 3.2% and and centrifuged at 1500 ×g for 15 min at 25°C; after blood withdrawn was mice were euthanized by anesthesia deepening. For APTT, a 50 *μ*L, of aliquot plasma was warmed to 37°C for 2 min, 50 *μ*L, of APTT reagent was added, of, after a 2 min incubation at 37°C, 0.25 M of CaCl_2_ was added, and the clotting time was determined. For the PT test, 100 *μ*L of CLOT PT reagent was incubated for 4 min at 37°C, and 50 *μ*L of plasma was added, triggering the reaction. These analyses were performed in triplicate, using the APTT and PT Kit CLOT BIOS diagnosis (CLOT, Brazil) in a CLOTimer coagulometer (CLOT, Brazil). To assay APTT and PT *in vitro*, mice plasma (45 *μ*L) was preincubated with 5 *μ*L of a different Rhombeobin (2, 5, 10, and 20 *μ*g/mL); then the standard analyses for APTT and PT were followed. To assay APTT and PT *ex vivo, *Swiss mice were injected i.v. with Rhombeobin (0.1 and 1 *μ*g/animal), and after 30, 60, and 90 min, blood was withdrawn and processed as described above.

### 2.13. Effects of Rhombeobin on the Mice Plasma Fibrinogen

The *ex vivo* test was performed according to Maruñak et al. [27] and modified as follows. Groups of mice with different exposure times (30, 60, and 90 min) and one control group, each one composite of four mice weighing 25 ± 2 g, were injected i.v. with 25 *μ*L of PBS (control group) or Rhombeobin (0.1 and 1 *μ*g/animal). After the times of exposure, blood samples and poor platelet plasma were obtained as described in Section 2.12. The fibrinogen content was determined using the Wiener diagnostic kit, and the results were expressed as percentage of the remaining fibrinogen compared with the control group.

### 2.14. Platelet Aggregation Assay

Washed platelets suspensions from mice were obtained according to Theakston and Reid [23]. Platelet aggregation was performed in an optical aggregometer (Chrono-log, Kordia Life Sciences, Leiden, Belgium) at 37°C with 400 *μ*L of washed platelets placed in glass cuvettes containing a disposable stir bar for constant stirring. Platelet aggregation was induced using ADP (20 *μ*M) or thrombin (0.05 U/mL) as agonist. The *ex vivo* effects of Rhombeobin over platelet aggregation were studied by injecting i.v. 25 *μ*L of PBS or enzyme (0.1 or 1 *μ*g/animal) in adult mice (25 ± 2 g). After 1 h, the mice were anesthetized, and washed platelets were obtained.

### 2.15. Statistical Analyses

Results were reported as mean ± SEM. The significance of differences among means was assessed by analysis of variance followed by Tuckey's test. The statistical analyses were made using the Origin 8 SR2 v8.0891 (B891; OriginLab Corporation, Northampton, MA, USA). Program *P* < 0.05 was considered statistically significant.

## 3. Results

**Figure 1:**
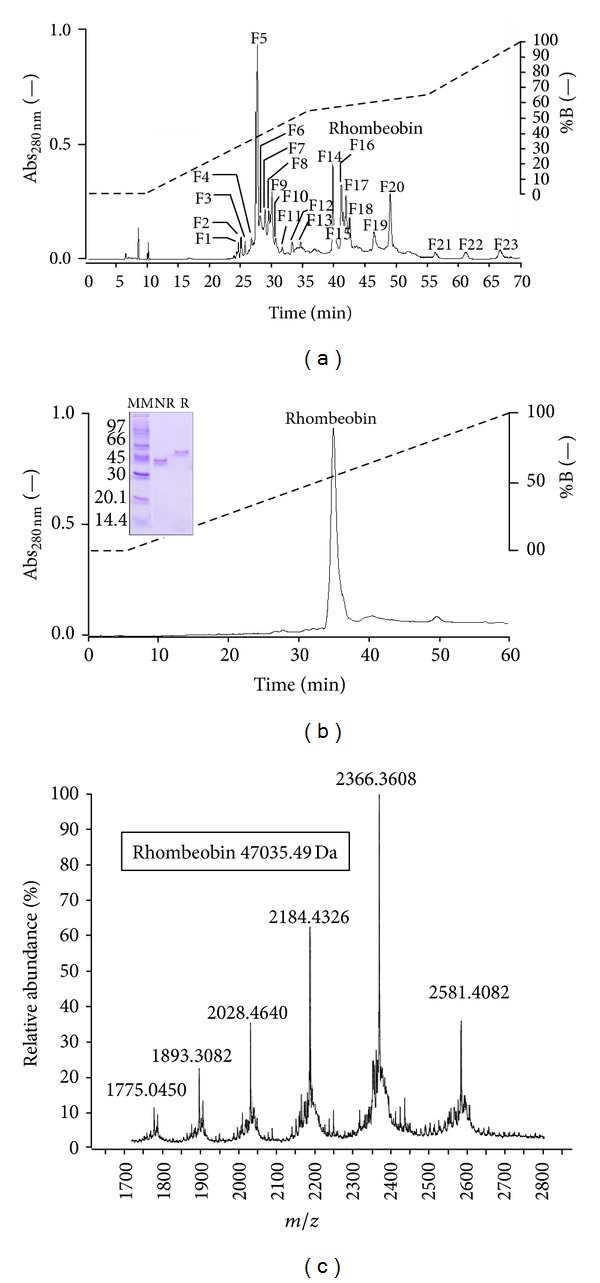
(a) Elution profile of *L. muta rhombeata* venom by the reversed phase HPLC on C8 Discovery BIO Wide Pore. Fraction 16 (F16) that contained both amidolytic and thrombin-like activities (Rhombeobin) from *L. muta rhombeata* venom is indicated. (b) Elution profile of F8 following RP-HPLC on C8 Discovery BIO Wide Pore. Insert: SDS-PAGE in nonreduced (NR) and reduced (R) conditions. (c) Molecular mass determination of the native Rhombeobin by nanoelectrospray tandem mass spectrometry, using a Q-Tof Ultima API mass spectrometer (MicroMass/Waters) with output mass range of 40.000–50.000 Da at a “resolution” of 0.1 Da/channel.

The fractionation of *Lachesis muta rhombeata* crude venom in an RP-HPLC system (C8 column) results in twenty-three main fractions named F1 to F23 (Figure 1(a)). Each fraction was assayed for amidolytic and coagulant activities, and both were found only in F-16 fraction. Rechromatography of F-16 fraction in an RP-HPLC, with a linear gradient, results in a single symmetric peak eluted with 57% of buffer B and a retention time of 36.23 ± 2.2 min (Figure 1(b)); SDS-PAGE showed that this fraction in nonreduced and reduced conditions is a single-chain protein, with a relative molecular mass of 45 kDa (Figure 1(b): insert). The protein homogeneity of F-16 was confirmed by ESI-MS, and it was shown that fraction F-16 is protein with a real molecular mass of 47035.49 Da (Figure 1(c)). 

**Figure 2:**
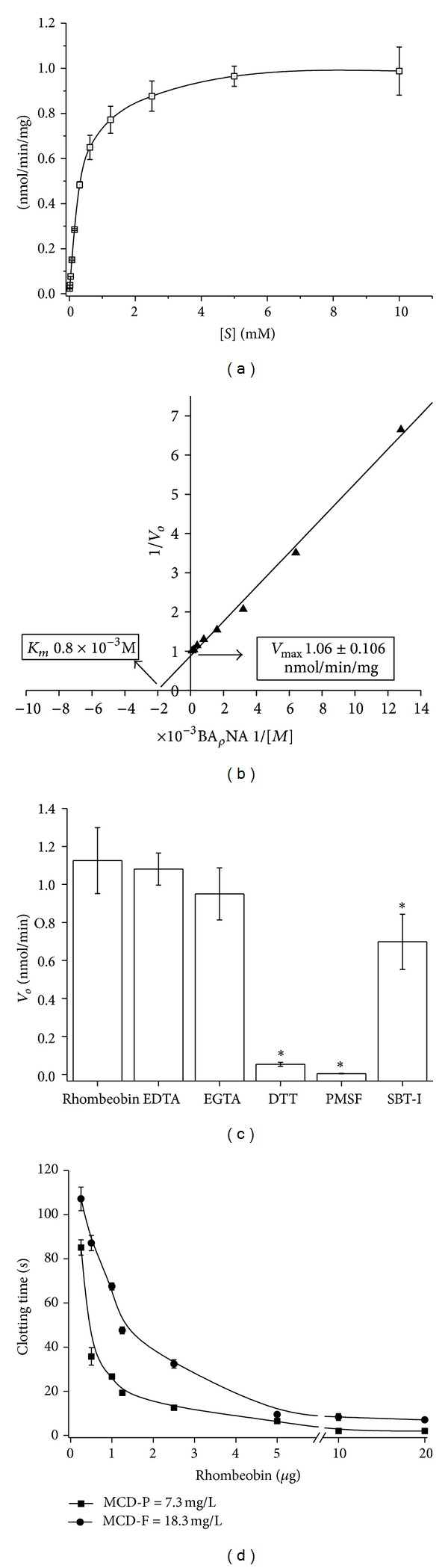
Enzymatic, kinetic properties and thrombin-like activity of Rhombeobin *L. m. rhombeata* venom. (a) The Michaelis-Menten curve. (b) The Lineweaver-Burk (double-reciprocal) plot. (c) Inhibition of the amidolytic activity by chelating agents (EDTA and EGTA), reducing agent (DTT), serineproteinase-specific inhibitors (PMSF), and soybean trypsin inhibitor (SBTI). (d) Minimum coagulant dose of Rhombeobin over citrated plasma (MCD-P) and fibrinogen bovine solution (2 mg/mL, MCD-F). The results of all experiments are the mean ± SEM of three determinations (*P* < 0.05).

The fraction F-16 was named Rhombeobin, and it showed an arginine amidase activity toward the chromogenic substrate BApNA with *K*_*m*_ and *V*_max⁡_ of 0.8 × 10^−3 ^M and 1.06 ± 0.106 nmol/min/mg, respectively (Figure 2(b)). The optimum temperature was between 40 and 50°C, and the remarkable stability in the pH was in the range of 7 to 10. The serineproteinase inhibitor PMSF and the reducing agent DTT completely abrogated Rhombeobin enzymatic activity, while SBT-I partially reduced (*P* = 0.0269) the enzymatic activity (Figure 2(c)). However, EDTA and EGTA did not show any effects (Figure 2(c)).

Rhombeobin is a strong thrombin-like enzyme since it can clot a bovine fibrinogen solution with an MCD-F of 18.3 mg/L of mice citrated plasma, with an MCD-P of 7.3 mg/L (Figure 2(d)).

**Figure 3:**
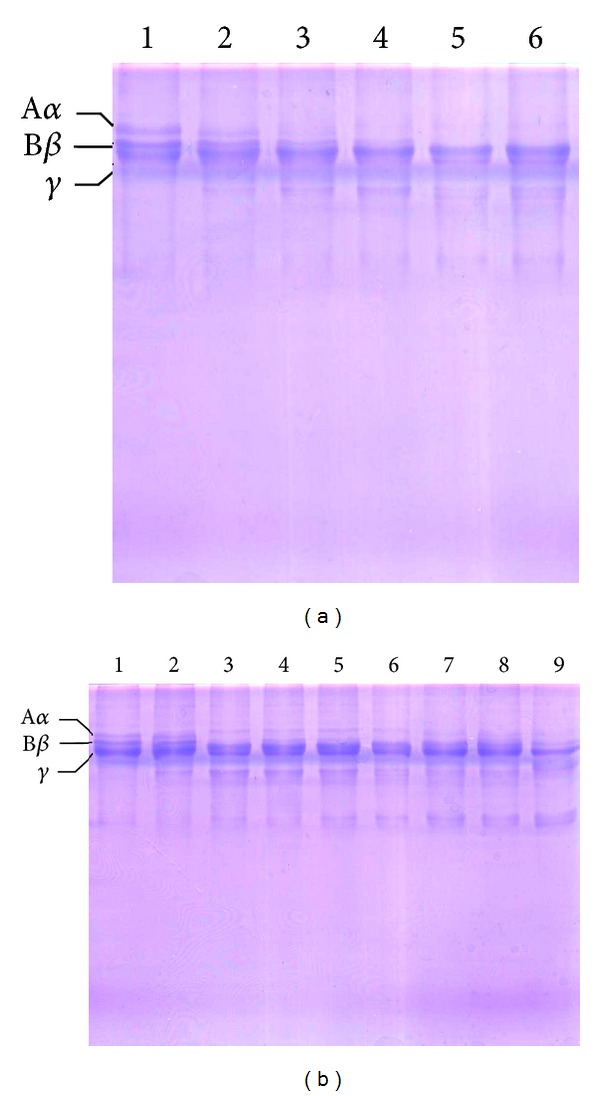
(a) Fibrinogenolytic activity of Rhombeobin at different concentrations: (1) bovine fibrinogen; (2) fibrinogen + Rhombeobin (0,625 *μ*g); (3) 1.25 *μ*g; (4) 2.5 *μ*g; (5) 5 *μ*g; (6) 10 *μ*g. (b) Fibrinogenolytic activity of Rhombeobin (2.5 *μ*g) at different incubation times: (1) bovine fibrinogen; (2) fibrinogen + Rhombeobin (5 min); (3) 15 min; (4) 30 min; (5) 1 h; (6) 3 h; (7) 6 h; (8) 12 h; (9) 24 h. The fibrinogenolytic activity was analyzed by SDS-PAGE (12.5%).

Rhombeobin showed a concentration and time-dependent fibrinogenolytic activity. This enzyme completely degraded fibrinogen *α*-chain with a concentration of 1.25 *μ*g with 15 min of incubation. Rhombeobin degraded fibrinogen *β*-chain at high concentrations and long incubation times; on the other hand, the enzyme did not show any activity over fibrinogen *γ*-chain (Figures 3(a) and 3(b)).

**Figure 4:**
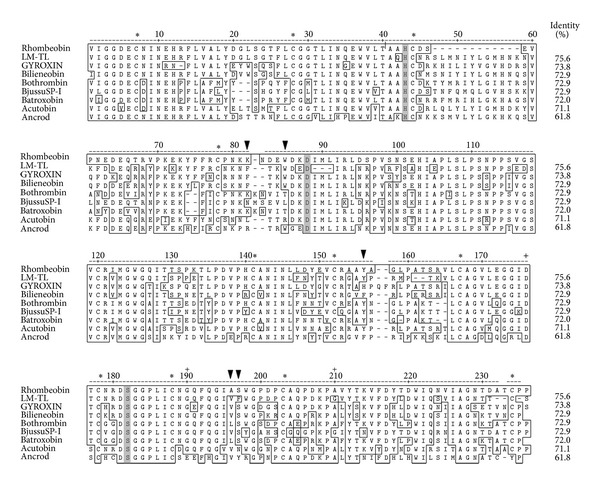
The amino acid sequence alignment of Rhombeobin with selected serine proteases sequences obtained from the BLAST protein data bank (PubMed/Medline). LM-TL, *Lachesis muta muta *[11]; gyroxin, *Crotalus durissus terrificus* [12]; bilineobin, *Agkistrodon bilineatus *[13]; bothrombin, *Bothrops jararaca *[14]; bjussuSP-I, *B. jararacusu *[15]; batroxobin, *B. atrox* [16]; acutobin, *Agkistrodon acutus *[17]; ancrod, *Agkistrodon rhodostoma* [18]. Numbering is according to ANCROD. Catalytic triad residues are shown in grey, and conserved cysteinve residues are shown by “∗”. Specificity sites and the residues forming the hydrophobic site are shown by “+” and “*▼*”, respectively, according to Castro et al. [19].

The protein digestion (Rhombeobin) with trypsin and protease SV-8, followed by LC/MS/MS, identified nine (T1–T9) and seven peptides (S1–S7), respectively. The deduced sequence and the measured masses of alkylated peptides are summarized in Tables 1 and 2, covering approximately 94% of the protein sequence. Each peptide was submitted separately to the SNAKE database using the protein search program BLAST-p. Using the position matches of the “*de novo”* sequenced peptides with homologous proteins present in the database, it was possible to deduce their original position on the unknown protein. Thus, it was deduced that peptides T-1, S-3, and T-8 contain the catalytic triad histidine, aspartic acid, and serine, respectively. Also, the digested peptides contain the twelve cysteine residues characteristic of the snake venom serineproteinase family. Rhombeobin showed homology (Figure 4) with other thrombin-like SVSPs from *Lachesis m. muta*, *Crotalus durissus terrificus*, *Agkistrodon bilineatus*, *Bothrops jararaca*, *Bothrops jararacussu*, *Bothrops atrox*, *Agkistrodon acutus,* and *Agkistrodon rhodostoma*, sharing high-sequence identity with them, especially with LM-TL from *Lachesis m. muta* (75.6%).

Rhombeobin induced “gyroxin syndrome” when injected into the mice tail vein at dose of 0.2 *μ*g/kg of bodyweight. The treated animals were progressively hypoactive over a period of 1–3 min, followed by a loss of the righting reflex, opisthotonos, and rotations around the long axis, lasting up to 30 minutes. All treated animals lost the ability to right themselves. Two hours following Rhombeobin injection, the animal's behavior returned to normal. Gyroxin syndrome was not observed with lower doses, in which the main effects were flaccid paralysis (mainly of inferior extremities) and hypoactivity.

**Figure 5:**
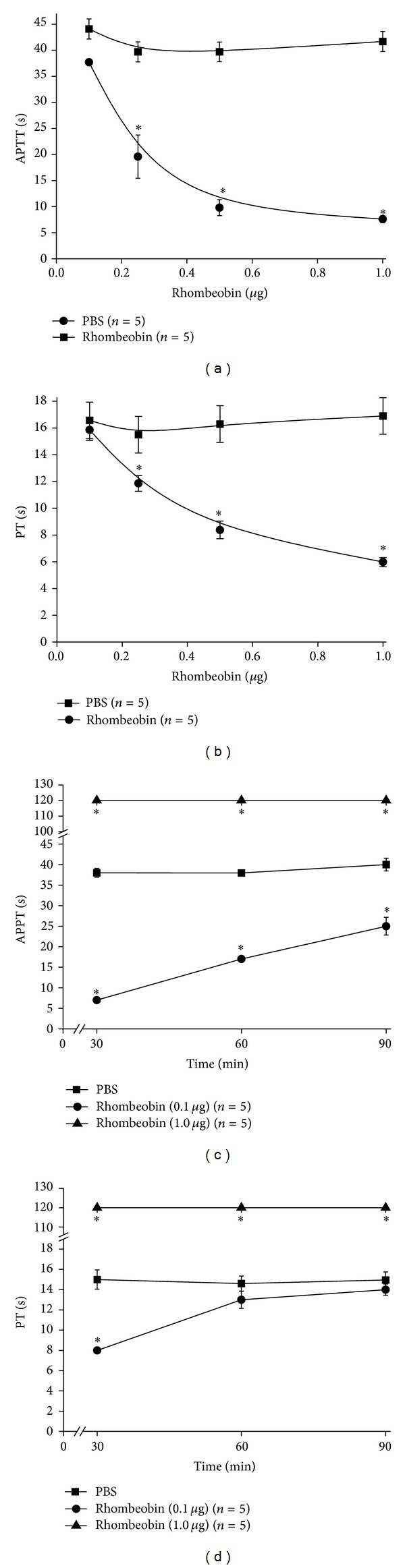
“*In vitro*” (a) activated partial thromboplastin time (APPT) and (b) prothrombin time (PT) blood tests of PBS (■) or different quantities of Rhombeobin (●) on mice citrated platelet poor plasma. *In vivo* time course effect on (c) APPT and (d) PT blood tests of male adult mice after i.v. injection of Rhombeobin (●) control mice received i.v injection of PBS (■) only. Results are expressed in seconds. Error bars indicate mean plus or minus standard deviation: “*in vitro*” (*n* = 3 for each group) and “*ex vivo*” (*n* = 5 for each group). Error bars indicate mean plus or minus SME (*n* = 5); ∗ means a significant difference (*P* < 0.05) compared with the control.

*In vitro,* Rhombeobin showed a strong procoagulant activity with a dose-dependent effect on citrated mice plasma, shortening significantly both APPT and PT tests (Figures 5(a) and 5(b)). 

Interestingly, *ex vivo*, Rhombeobin treatment produced both anticoagulant and procoagulant effects. The i.v. injection, with 1 *μ*g of protein, renders unclottable blood after 30 minutes of exposure. This effect was sustained through the whole time of the experiment (Figures 5(c) and 5(d)). On the other hand, Rhombeobin shortened the APTT and PT tests in mice treated with a dose ten times lower; this was a rapid and sustained effect on APTT test (Figure 5(c)); although on PT test the shortening effect was rapid, one hour after injection this effect was not observed (Figure 5(d)).

**Figure 6:**
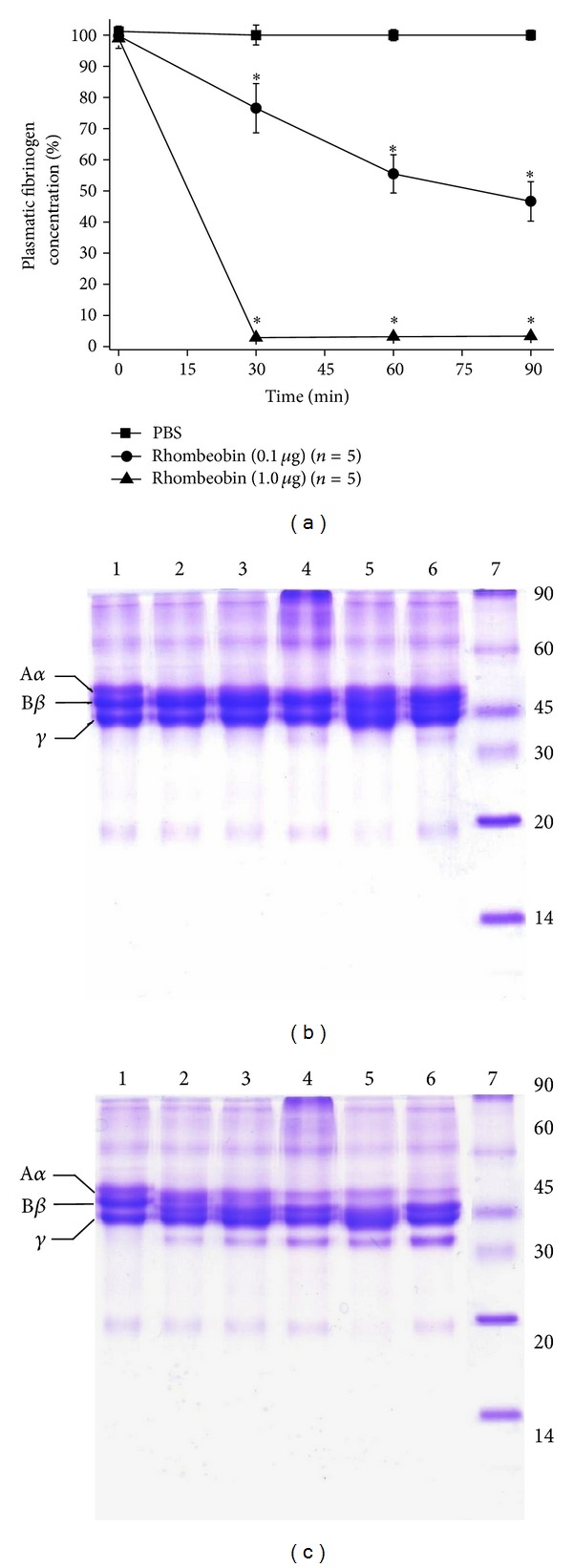
(a) Relative levels of mouse plasma fibrinogen after intravenous injection of PBS (■) or Rhombeobin: 0.1 *μ*g (●) and 1 *μ*g (▲). Values were expressed as % compared with the control. Rhombeobin fibrinogenolytic activity at different incubation times: (1) bovine fibrinogen; (2) fibrinogen + Rhombeobin (5 min); (3) 15 min; (4) 30 min; (5) 1 h; (6) 2 h; 0.1 *μ*g (b) or 1 *μ*g (c) of enzyme mixture with bovine fibrinogen solution (2 mg/mL) and analysis by SDS-PAGE (12.5%); MW: molecular markers. Error bars indicate mean plus or minus SME (*n* = 5); ∗ means significant difference (*P* < 0.05) compared with the control.

Mice plasma fibrinogen levels were determined after Rhombeobin i.v. injection. At 1 *μ*g dose, the protein showed a strong defibrinogenating effect, with no detectable plasma fibrinogen levels after thirty minutes after injection (Figure 6(a)). On the other hand, with a dose ten times lower, only partial reduction of plasma fibrinogen levels was observed (Figure 6(a)). These results corroborate with the fibrinogenolytic activity on bovine fibrinogen analyzed by SDS-PAGE. While 0.1 *μ*g of the protein lightly degraded fibrinogen *α*-chain (Figure 6(b)), 1 *μ*g, after 90 minutes of incubation, almost completely degraded fibrinogen *α*-chain, producing fibrinopeptide A (Figure 6(c)).

**Figure 7:**
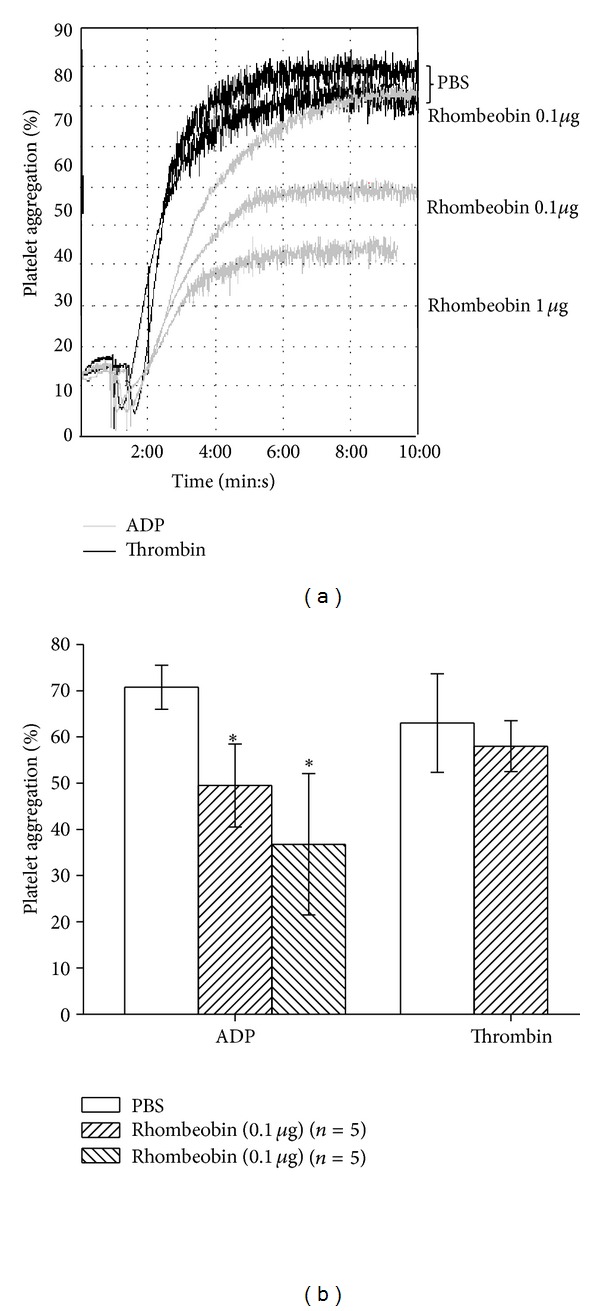
“*In vivo*” effect of Rhombeobin on platelet aggregation. (a) Representative recordings showing the platelet aggregation of mice washed platelet suspension with ADP (grey line) or human thrombin (black line) one hour after i.v. injection of the enzyme (0,1 and 1 *μ*g/animal). Control animals received PBS only. (b) Bar representation of platelet aggregation. Error bars indicate mean plus or minus standard deviation (*n* = 5 for each group); *P < 0.05.

When mice were treated with Rhombeobin i.v. at 0.1/animal and after one hour platelet suspensions were obtained, a significant reduction (*P* = 0,03063) of the platelet aggregating response to ADP as agonist was observed. However, no effect over the platelet aggregating response was observed when Thrombin was used as agonist (Figures 7(a) and 7(b)). 

## 4. Discussion

This work reports an efficient and simple procedure for the purification of LMR-47: a thrombin-like/gyroxin-like enzyme from *Lachesis muta rhombeata* venom, which we renamed as Rhombeobin. In our approach, using a simple chromatographic step based on RP-HPLC showed that the *L. m. rhombeata* venom can be separate in twenty-three main fractions. From all of them, only fraction sixteen presents amidolytic and thrombin-like activities. The purification of thrombin-like enzymes from the venom is usually made using methods based on molecular-size exclusion followed by either ion-exchange or affinity-binding (benzamidine- or arginine-Sepharose) chromatography [8], but this approach requires several steps, considerable time, and loss of material by the dialysis procedures prior to obtaining a lyophilized sample. Asingle chromatographic step using HPLC systems provided more purified protein samples than other conventional methods reducing time and material loss. de-Simone et al. [14] reported the purification of LMR-47 (Rhombeobin) with a single affinity chromatography step on HPLC system using two columns in tandem with the same ligand; although the authors were able to obtain a purified protein, the purification method was rather complicated by the time require and did not improve the protein recovery observed with conventional methods [9]. In comparison, with the purification procedure described here, we were able to recover near 5% of the purified protein (data not shown), twice more efficient in terms of protein recovery.

The molecular homogeneity of Rhombeobin was assessed by RP-HPLC (Figure 1(b)) showing a single and symmetric peak with elution and retention time typical for the snake venom serineproteinase family [28–30]. The SDS-PAGE analysis (Figure 1(b), insert) showed a single band with an identical electrophoretic mobility and relative molecular mass of ~45 kDa shown, by LMR-47 [9]. ESI-MS analysis showed that the molecular mass of Rhombeobin was 47035.49 Da.

Rhombeobin showed a high amidolytic activity upon BApNA with a Michaelian enzymatic behavior (Figure 2(a)). The calculated values of *K*_*m*_, *V*_max⁡_, and  *k*_cat_ (Figure 2(b)) were similar to those exhibited for LMR-47 over the same substrate [9]. As with other snake venom serine proteinases [31–33], Rhombeobin loses all enzymatic activity at high temperatures. Interestingly, this enzyme showed a broad range of optimum pH; the maximal enzymatic activity remained unaltered along of neutral and mild basic conditions (pH 7–10). The high enzymatic activity observed for Rhombeobin at the range of pH has been described for other snake venom serine proteinases with thrombin-like activity like bothrops protease A (*Bothrops jararaca*) [34]. PMSF completely inhibited enzymatic activity of Rhombeobin. On the other hand, the partially inhibitory effect of Kunitz-type inhibitor SBT-I over the enzymatic activity of this enzyme has been described for other snake venom serine proteinases, although complete inhibitions were achieved, in some cases, at high concentrations [8]. As a thrombin-like enzyme, Rhombeobin showed a high clotting activity upon bovine fibrinogen, and its activity was higher over citrated plasma (Figure 2(d)). This enzyme showed an *α*-fibrigenonolytic behavior when incubated with bovine fibrinogen, and it degraded fibrinogen *β*-chain at higher doses and prolonged incubation times (Figures 3(a) and 3(b)). The thrombin-like activity and the pattern found on the fibrinogenolytic activity of Rhombeobin were similarly shown by SVSP LMR-47 [9]. 

The biochemical, enzymatic, and biological data obtained in this work strongly suggest that Rhombeobin is the same protein LMR-47 thrombin-like/gyroxin-analog characterized by Aguiar et al. [9]. According to the Scientific and Standardization Subcommittee of the International Society on Thrombosis and Haemostasis [11], we propose to rename LMR-47 as Rhombeobin, which is the result of the combination of the subspecies name *rhombeata* with the suffix “obin”. 

The results obtained from the sequences provided several peptides derived of trypsin (Table 1) and SV-8 (Table 2) enzyme digestion; from these peptides, we were able to deduce almost the entire Rhombeobin protein sequence. Furthermore, the peptides named T-1, S-1, and S-2, combined, contain a 46 N-terminal amino acid sequence which showed 100% identity with the 30 N-terminal of LMR-47 [9], confirming that Rhombeobin and LMR-47 are the same protein. 

The homology study (Figure 4) showed that Rhombeobin shared a high degree of sequence identity with SVSP with thrombin-like activity [12, 13, 15–18, 28, 35] especially with LM-TL (*Lachesis muta muta*). Detailed analysis showed small but important differences between Rhombeobin and LM-TL. In both enzymes, the primary (D176) and secondary (G199) specificity sites are conserved, while the tertiary specificity site (position 210) in LM-TL is a Gly residue, while in Rhombeobin it is an Ala residue. Another difference is observed in part of the hydrophobic site [19], LM-TL has Phe, Val, and Phe residues in positions 82, 196, and 197, respectively, while Rhombeobin shows Lys, Ala, and Ser in the same positions. These substitutions suggest that these proteins could have differences in their affinity for natural and synthetic substrates [36]. Also, there are some modifications in the region called “90 Loop” (position 79 to 85 in our numbering), in which Phe82 and Trp85 are responsible for the resistance of LM-TL to the inhibitory effect of Kunitz-type inhibitors by obstruction of the inhibitor binding region and sterically hindering other residues (Lys80, Arg180, and Ser233) [19]. The substitution of Phe82 by Lys82 in Rhombeobin could have allowed better interaction with the binding site of SBT-I inhibitor as suggested by the results of the inhibitory studies (Figure 2(c)). 

In general, snake venom serine proteinases (SVSPs) affect the coagulation cascade by activation or proteolytic degradation of specifically coagulation factors [8, 36]. A number of snake venom serine proteinases with thrombin-like activity showed procoagulant activity “*in vitro*” by direct activation of factor V [37–39], factor VIII [28, 40], and factor X [41]. *In vitro*, Rhombeobin acts as a procoagulant SVSP shortening both APPT and PT tests (Figures 5(a) and 5(b)). These results suggest that this enzyme could act over the common coagulation pathway, likely by activating coagulation factor V and/or factor X. However, the fact that Rhombeobin induces a more marked shortening of APPT than PT test could be an indicator of activation of other specific intrinsic pathway factors. 

Venom-induced consumptive coagulopathy or VICC [42] is characterized by activation of the coagulation pathway and consumption of coagulation factors, mainly fibrinogen, resulting in multiple factor deficiencies in snakebitten patients [42–44]. In laboratory, it is characterized by the alteration of basic clotting tests like WBTC, APPT, and PT tests [42, 43]. In this regard, Rhombeobin (0.1 *μ*g/animal) activates both the intrinsic and extrinsic coagulation pathways (Figures 5(c) and 5(d)), but it does not induce defibrinogenation (Figure 6(a)) because, at this concentration, Rhombeobin did not effectively degrade fibrinogen (Figure 6(b)). However, with 1 *μ*g/animal, Rhombeobin rendered mice blood unclottable (Figures 5(c) and 5(d)), by activating the blood coagulation factors and mainly by a rapid and sustained defibrinogenation (Figure 6(a)) due to a proteolytic degradation (Figure 6(c)). Thus, Rhombeobin appears to be a key player in the induction of the severe coagulopathy induced by *L. m. rhombeata *venom [6]. 

Snake venom serine proteinases also act on platelet-rich plasma or washed platelet suspensions promoting platelet-aggregation and platelet release reactions [8, 36]. This activation is mediated by the proteolytic activation of the membrane receptors PAR1 and PAR4 [45], or by interacting with glycoprotein GPIb or GPIIbIIIa [41]. 

However, when Rhombeobin was injected i.v. in adult mice, we observed a reduction on platelet aggregation in response to ADP (20 *μ*M, Figures 7(a) and 7(b)) as agonist. This impairment of platelet function was found to be well correlated with a decrease in plasma fibrinogen concentration, an effect previously described for other SVSPs with thrombin-like activities [46]. Interestingly, Rhombeobin did not show any effect over the activity of thrombin as platelet aggregation agonist.

## 5. Conclusion

Our results showed that venom components, like Rhombeobin, can evoke a venom-induced consumptive coagulopathy by itself, mainly through procoagulant effect mainly through the intrinsic and common pathway that involves catalytic activation of coagulation factors and the enzymatically hydrolyzing plasma fibrinogen. Thus, Rhombeobin has a main role in the coagulopathies induced by *Lachesis muta rhombeata* snake venom. The mechanisms by which Rhombeobin interacts with platelet membrane receptors *in vivo* are different from those described in the literature, and, therefore, necessary additional studies for better understanding of the “*in vivo*” mechanism of this enzyme that delays the thrombus formations are needed.

## Figures and Tables

**Table 1:** Measured molecular masses and deduced amino acid sequences obtained by ESI-Q-Tof-MS/MS based on the alkylated tryptic peptides of Rhombeobin.

Peptide	Position	Amino acid sequence	Measured mass (Da)
T-1	1–13	VI/LGGDE*C*NI/LNEH**R**	1497.6263
T-2	60–71	VPNEDEQT**K**YP**K**	1474.7011
T-3	72–80	E**K**YFFR*C*PN**KK**	1463.7229
T-4	85–93	WDKDI/LMI/LI/L**R**	1188.6088
T-5	94–121	I/LDSPVSNSEHI/LAPI/LSI/LPSNPPSVGSV*C * **R**	2915.3930
T-6	122–132	I/LMGWGQTI/LTSP**K**	1317.6720
T-7	160–183	VI/L*C*AGVI/LEGGI/LDT*C*N**R**	1732.7533
T-8	184–217	DSGGPI/LI/LCNGQFQGI/LASWGPDPCAQPD**K**PAVYT**K**	3630.5924
T-9	218–239	VFDYTDWI/LQNI/LI/LAGNTDAT*C*PP	2496.0740

The peptides were separated by RP-HPLC and were sequenced by mass spectrometry. *C* = alkylated cysteine; lysine and arginine residues shown in bold were deduced on the cleavage and missed cleavage by trypsin and SV-8. All molecular masses are reported as monoisotopic.

**Table 2:** Measured molecular masses and deduced amino acid sequences obtained by ESI-Q-Tof-MS/MS based on the alkylated SV-8 peptides of Rhombeobin.

Peptide	Position	Amino acid sequence	Measured mass (Da)
S-1	1–11	VI/LGGDECNIN**E**	1218.5202
S-2	12–36	HRFI/LVAI/LYDGI/LSGTFI/L*C*GGTI/LI/LNQ**E**	2780.3842
S-3	37–48	WVI/LTAAH*C*DS**E**	1287.5534
S-4	66–72	QTRYPK**E **	920.4763
S-5	73–82	KYFFR*C*PNKKND**E**	1744.8213
S-6	134–150	TI/LPDVPH*C*ANI/LNI/LI/LDY**E**	1982.8704
S-7	151–174	V*C*RAAYAGI/LPATSRVI/L*C*AGVI/L**E**	2333.1693

The peptides were separated by RP-HPLC and were sequenced by mass spectrometry. *C* = alkylated cysteine; glutamic acid residues shown in bold were deduced on the cleavage *Streptoccocus aureus * SV-8. All molecular masses are reported as monoisotopic.
